# Start-to-end modelling of laser-plasma acceleration, beam transport and dose deposition of very high-energy electrons for radiotherapy

**DOI:** 10.1038/s41598-026-49116-8

**Published:** 2026-04-24

**Authors:** Rajakrishna Kalvala, Anton Golovanov, Arnaud Courvoisier, Tomer Friling, Eyal Kroupp, Lidan Grishko, Victor Malka

**Affiliations:** Department of Physics of Complex Systems, Weizmann Institute of Science, Rehovot, 7610001 Israel

**Keywords:** VHEE radiotherapy, Laser-plasma acceleration, GEANT4, Particle-in-cell, Cancer, Oncology, Physics

## Abstract

The proposed radiotherapy using very high-energy electron (VHEE) beams generated by a laser-plasma accelerator has garnered significant interest due to its dose distribution capabilities and potential to address limitations of traditional photon-based radiotherapy. To explore the feasibility of such an approach, we develop a start-to-end simulation workflow to model VHEE radiotherapy from the electron source through the beamline to dose delivery in the target. The presented study uses the parameters of OONA, the commercial <1.3 J, <25 fs pulse duration laser recently installed at the Weizmann Institute of Science. Through particle-in-cell simulations of laser-plasma interaction, realistic electron beams are obtained. A beamline consisting of quadrupoles, a collimator, and dipoles is then used to collimate and filter the beams and arrange them into a beam array. Using GEANT4 simulations, we calculate the dose deposition in water phantoms and heterogeneous phantoms with bone inserts. Multifield irradiation setup and the dose distribution at the isocenter through different incidence angles are studied, simulating multi-angle conformal delivery. Our findings demonstrate that polychromatic VHEE beams generated from laser-plasma accelerators, when delivered through such a beamline, can achieve favorable dose distribution for reaching areas deep inside the phantom. This study highlights the potential of the developed start-to-end workflow for exploring and optimizing LPA-generated VHEE radiotherapy, paving the way for further research and potential clinical implementation.

## Introduction

Radiotherapy remains a cornerstone of cancer treatment, with approximately 50% of all cancer patients receiving some form of radiation therapy during their treatment course^1,2^. Conventional photon radiotherapy remains limited in its ability to effectively treat deep-seated tumours while adequately sparing surrounding healthy tissues. Photon beams result in unavoidable entrance and exit dose deposition beyond the target volume, constraining treatment optimization^3^. In comparison, proton therapy offers superior dose conformity through its characteristic Bragg peak, but its widespread implementation is hindered by high infrastructure costs and accessibility challenges, with fewer than 200 centres worldwide serving the global cancer population^4,5^. This substantial gap between treatment availability and clinical needs^6,7^ underscores the urgent necessity for innovative radiotherapy modalities that can combine the advantageous dose deposition characteristics of particle therapy with the accessibility and cost-effectiveness of conventional systems.

To improve the effectiveness of radiation oncology and enhance patients’ health-related quality of life, radiotherapy must be capable of inducing cancer cell death while preserving healthy tissues^3^. Current studies highlight very-high energy electron (VHEE) radiotherapy as a promising approach to achieve these goals^8–13^. VHEE beams in the 150–250 MeV range were first proposed by DesRosiers et al. as a novel radiotherapy modality allowing the treatment of deep-seated tumours^14^. Since then, several studies have emphasized the advantages of VHEE use in radiotherapy. Unlike conventional electron therapy with 5–25 MeV electrons, which is typically limited to treating superficial tumours due to their low penetration depth of 1–7 cm, VHEE beams can effectively reach deep-seated tumours at depths of 10–15 cm with millimeter precision^15,16^. Combined with the insensitivity of VHEE treatment to anatomical heterogeneities, it represents a significant advancement in radiation oncology, potentially expanding the therapeutic options available for treating complex and challenging malignancies^17–20^. Moreover, to increase the conformal dose delivery to deep targets, VHEE beams of more than 100 MeV were found to be desirable for intensity-modulated radiotherapy^21^. Comparative treatment planning studies between conventional radiotherapy modalities and VHEE have shown lower spinal cord dose in the pediatric brain case and lower brain stem dose in the VHEE case^15,17,22^. Additionally, VHEE beams can be electromagnetically scanned or focused with high precision^10,23^, potentially enabling accurate dose delivery to the tumour volume while minimising exposure to surrounding healthy tissues^11,24,25^.

VHEE beams have also started gaining wider interest with the advent of laser-plasma accelerators (LPAs) that can generate ultra-high accelerating fields, significantly reducing the size and cost of accelerator facilities compared to conventional linear accelerators^26–29^. Recent advancements in LPA technology have significantly improved electron beam quality and succeeded in achieving low energy spread and enhanced shot-to-shot stability in the energy range suitable for VHEE radiotherapy^30–32^. Even though in several aspects the quality of the beams remains below the level of conventional accelerators, these advancements make LPAs favorable candidates for future clinical applications^12,18,33–36^. Unique properties of LPA-generated electron beams compared to beams from conventional linacs require specific beamline designs^11,13,24^and paying attention to the influence of the energy spread and angular divergence on dose delivery. This poses an additional challenge for numerical simulations, as a rigorous investigation must include generation of an electron beam in a LPA, its propagation through the beamline, and the following interaction with the phantom. However, previous simulations of LPA-based VHEE used approximate models of LPA-generated beams instead^16,33,37^. Such an approach cannot accurately take into account all the properties of LPA-produced beams, such as different divergence for different energies and transverse asymmetries, which are crucial for their interaction with the beamline, so it can be used only for qualitative estimates of VHEE radiotherapy. Additional challenges include the development of appropriate dosimetry techniques for accurately measuring and monitoring VHEE beams, managing secondary radiation (particularly neutron production), and designing compact, cost-effective systems suitable for clinical environments^18,38^.

In this study, we employ a start-to-end computational approach to evaluate the potential of LPA for generating VHEE beams in the context of radiotherapy. Particle-in-cell (PIC) simulations are used to model the interaction between an intense laser pulse and a gas target, optimizing the conditions for producing suitable electron beams. Simulations are performed for different gas target parameters with different target energies of the accelerated beams. The generated beam is then processed through beamline simulations, including filtering and collimation, to refine its energy spectrum and spatial characteristics for therapeutic applications. Finally, GEANT4 simulations using these PIC generated beams are conducted to analyze dose deposition patterns in both water and heterogeneous phantoms. In these simulations, we consider an interaction geometry in which a 7 $\times$ 7 array of LPA-produced beams steered by dipole magnets converges at the same spatial point. This geometry showcases the full capability of using the start-to-end simulation approach and is shown to reduce the entrance dose and increase the longitudinal localization of the dose distribution. The possibility of using this array configuration in a multi-field irradiation configuration is also studied. This start-to-end approach enables the detailed study of LPA-generated VHEE beam radiotherapy and paves the way towards integrating such beams into clinical treatment planning systems.

## Results

The numerical study of dose deposition in phantoms is critical for optimizing radiation therapy techniques, as accurate simulations help to predict dose distributions and enhance treatment efficacy. Here we establish a start-to-end simulation workflow and investigate how different electron beam parameters that are generated in laser-plasma accelerators and propagated through a beamline affect the 3D dose deposition in a water-equivalent phantom and a heterogenous phantom with a bone insert of 2 cm using PIC and GEANT4 simulations.

### LPA stage

To simulate the generation of an electron beam in a LPA, we perform PIC simulations of the interaction of a laser pulse with a gas jet using FBPIC, a spectral PIC code with angular mode decomposition widely used for simulations of LPAs^39^. PIC simulations are based on solving Maxwell’s equations for the electromagnetic fields of the laser pulse and the plasma wakefield together with the equations of plasma particle motion and can capture all fundamental physics of the laser-plasma interaction, allowing for a first-principle reconstruction of LPA experiments.

In the simulations, a 25 fs (intensity FWHM) laser pulse with energies of either 0.3 J or 0.5 J (corresponding to the peak power of 11 TW or 19 TW, respectively) is focused down to a spot size of 12 $\upmu$m onto a jet of air or a helium–nitrogen mixture. The parameters of the laser pulse correspond to the capabilities of the OONA laser system at the Weizmann Institute of Science in Israel. Air is used as a widely available gas whose usage could potentially reduce the cost of a commercial LPA device, while the helium–nitrogen mixture serves as a reference gas due to its common use in LPA experiments. The jet is represented in the simulations as a partially pre-ionized plasma with a trapezoidal longitudinal profile.

**Fig. 1:**
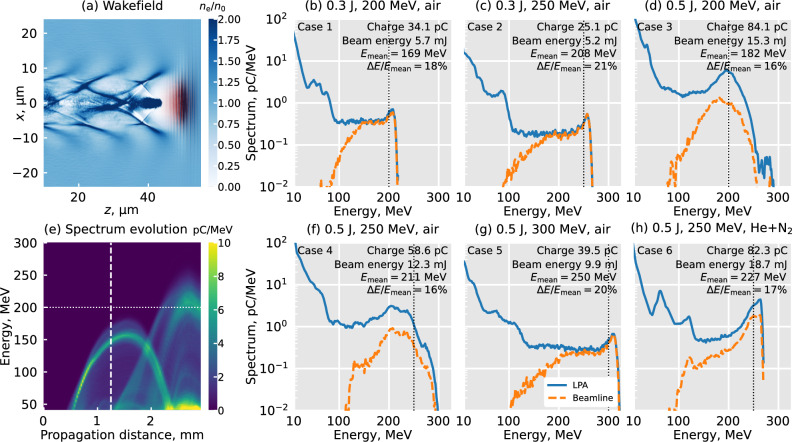
(**a**) Electron number density distribution (normalized to the expected background electron plasma density $n_0$) in a laser-driven wakefield corresponding to the case (**d**). The laser pulse intensity is shown with the red color. (**b**–**d**, **f**–**h**) Energy spectra of electron beams after the laser-plasma accelerator (solid lines) and after the beamline (dashed lines) for different laser energies (0.3 J and 0.5 J), different target energies (200 MeV, 250 MeV, 300 MeV) of the electron beam, and different gas mixtures (air or a helium–nitrogen mixture). The total charge, the total energy, the mean electron energy $E_\textrm{mean}$, and the relative energy spread $\Delta E/E_\textrm{mean}$ for the beam after the beamline are specified for each case. The vertical dotted lines show the target electron energy. (**e**) The evolution of the beam electron spectrum with the laser propagation distance. The case corresponds to the final spectrum shown in (**d**). The horizontal dotted line shows the target electron energy, while the vertical dashed line shows the distance corresponding to the wakefield snapshot in (**a**).

Upon entering the gas target, such a short and high-power laser pulse almost fully ionizes the gas and drives a nonlinear wakefield in the resulting underdense plasma. An example of such a wakefield with the typical “bubble” structure behind the laser driver is shown in Fig. 1(a). The electrons are then injected into the plasma wakefield through ionization injection, a process when electrons from lower shells of nitrogen and oxygen are ionized near the peak field of the laser pulse already inside the wakefield, which ensures their trapping^40^. In the case of the helium–nitrogen mixture, helium serves as the background gas responsible for the creation of the plasma, and the amount of the injected electrons can be controlled by changing the concentration of nitrogen which is typically a few percent. Air always provides very strong ionization injection as both of the main gas components (nitrogen and oxygen) have lower shells which are ionized only inside the laser pulse at the considered intensity. Ionization injection was chosen as the main injection mechanism due to the ease of its practical implementation—which requires only choosing a gas containing nitrogen or oxygen—and the overall robustness of the scheme. The parameters of the gas jet (its position relative to the laser focus, the gas density, and the length of the gas profile) are chosen to produce electron bunches with a high charge and target energies of 200 MeV, 250 MeV, and 300 MeV through a parameter optimization procedure described in the Methods section.

The resulting spectra from PIC simulations are shown with solid lines in Fig. 1(b–d, f–h). In all cases, we observe electron bunches with a wide energy spectrum and a high number of low-energy electrons, which is typical for the ionization injection scheme. However, the spectra have noticeable peaks near the target energy. Figure 1(e) shows an example of the evolution of the electron spectrum during the laser propagation through the gas for the final spectrum from Fig. 1(d). It demonstrates the injection and acceleration of the electron beam to the target energy. The results show that obtaining electron beams with a desireable energy for VHEE radiotherapy is possible by tweaking the parameters of the laser–gas interaction even for moderate laser energies, either when using a more conventional helium–nitrogen mixture or when using air.

### Beam transport line

Immediately at the exit from the LPA stage, the electron beam has a very small transverse size (several $\upmu$m) but a relatively high divergence (typically several mrad). To improve the electron beam spectrum by filtering out low-energy electrons as well as to collimate the beam, we utilize a beam transport line.

**Fig. 2:**
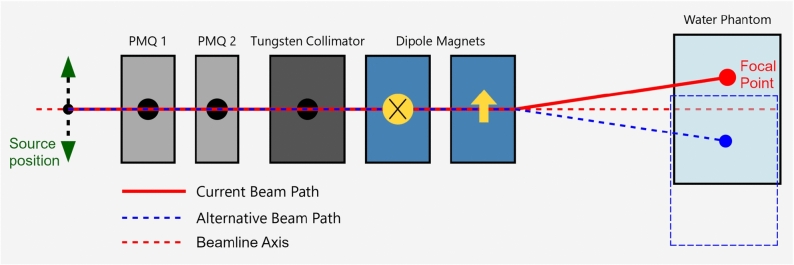
A schematic of the beamline with two permanent magnet quadrupoles (PMQs), a collimator, and two dipole magnets (the cross and up arrows distinguishes the perpendicular to each other dipole magnetic field directions).

The design of the beam transport line from the LPA stage to the phantom is illustrated in Fig. 2. The beam transport line consists of two permanent magnet quadrupoles (PMQs) for the collimation of the electron beam^41,42^, a collimator to prevent highly divergent low-energy electrons from reaching the phantom, and two dipole magnets to steer the beam to the desired angle in two perpendicular directions. The quadrupoles have a focusing gradient of 545 T/m, lengths of 2 cm and 1.5 cm, respectively, and an inner radius of 3 mm. They provide focusing in two different perpendicular directions, respectively, with the first quadrupole providing focusing in the laser polarization direction *x*. Their position is adjusted to ensure proper collimation of electrons at the target energy determined by the LPA devices and corresponding to the target energies in Fig. 1 and therefore changes for different target energies. The collimator is located 15 cm away from the center of the second quadrupole and has a pinhole with a radius of 1 mm. The dipoles have a length of 1.5 cm and an adjustable magnetic field of up to $\pm {2}\,{\mathrm{T}}$ each, which can be achieved using compact pulsed dipoles based on Helmholtz coils driven by a capacitor-discharge circuit delivering kiloampere-level currents.

**Fig. 3:**
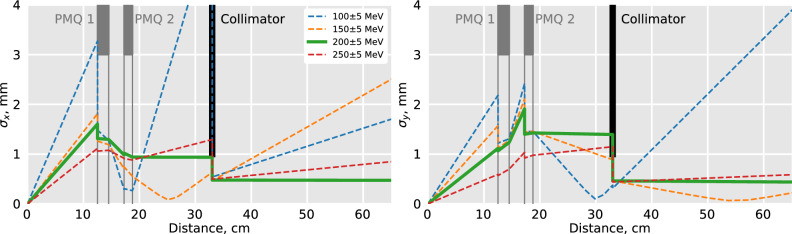
Evolution of the transverse sizes $\sigma _x$ and $\sigma _y$ of different energy components of an electron beam as it propagates through the beamline. The evolution of the component corresponding to the design energy of the beamline (200 MeV) is highlighted with a solid thicker line. The propagation distance is measured from the source. The permanent magnet quadrupoles (PMQ 1 and PMQ 2) are plotted with dark gray lines with color filling above their inner radius of 3 mm, and the collimator pinhole with the radius of 1 mm is shown with a thick black vertical line. The transverse size is defined as the standard deviation, $\sigma _x =\sqrt{\overline{x^2} - \overline{x}^2}$, and averaging is performed over beam electrons within a certain energy range, e.g. $200\pm 5$ MeV. The considered beam corresponded to Fig. 1(d).

The evolution of the transverse size of different energy slices of a beam corresponding to Fig. 1(d) propagating through the simulated beamline without steering by the dipoles is shown in Fig. 3. The beamline is tuned for a particular energy corresponding to the target electron energy in every case in Fig. 1 (see the Methods section for the beamline parameters). Any electrons that are farther away from the beamline design energy are scattered by the quadrupoles and most of them are consecutively filtered out by the collimator device positioned after the quadrupoles. Lower energy electrons from LPAs typically experience larger divergence and can scatter even before reaching the quadrupoles. However, the electrons with the energy corresponding to the design energy of the beamline (shown with the thick solid lines in Fig. 3) experience collimation, and most of them successfully propagate through the collimator and keep the same transverse size after it, making them usable for dose delivery. The resulting beam radius at the phantom entrance is on the order of the collimator aperture (1 mm).

Dashed lines in Fig. 1 show the energy spectra of electron beams after the collimator. In all cases, we observe a very significant reduction in the low-energy part. In some cases, the reduction happens also for the target energy, owing to high divergence of a beam from the LPA stage which results in the collimated beam size being larger than the pinhole radius and some of the collimated electrons being filtered out.

**Fig. 4:**
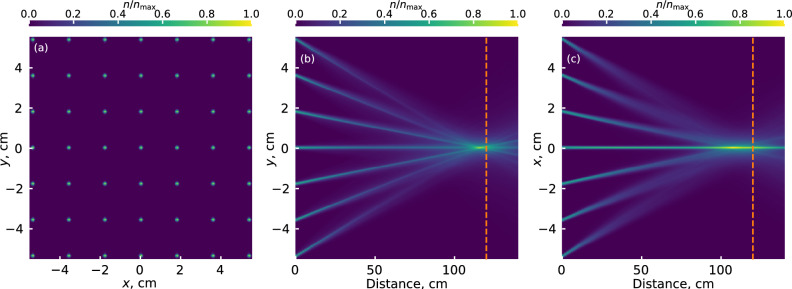
The initial transverse electron density distribution (**a**) and the evolution of the electron density projections to the *y* (**b**) and *x* (**c**) directions with the propagation distance for the propagation of a $7 \times 7$ array of beams in vacuum. The used beam corresponded to Fig. 1(d). The vertical dashed lines show the expected conversion point (in the absence of the phantom) 1.2 m away from the beamline’s exit.

The steering capability of the two dipoles located after the collimator is used to form a $7 \times 7$ array of beams converging at the same point (Fig. 4). In practice, it can be achieved either by moving the source and the beamline assembly or by moving the phantom in the transverse direction after each laser shot (or several consecutive shots with the same angle), while adjusting the magnetic field of the dipoles to ensure they pass through the same point in the phantom. Enabling motion of the source only in one plane compared to a more complex rotating gantry can possibly reduce the cost of the LPA-based radiotherapy device. The required magnetic fields of the dipoles are calculated for the target bunch energy. The maximum dipole strength determines the angular aperture of the array as the steering angle is calculated as $\theta _\textrm{max} \approx e c B_\textrm{max} L/\varepsilon$, where *e* is the elementary charge, *c* is the speed of light, $B_\textrm{max} = {2}\,{\textrm{T}}$ and $L = {1.5}\,\textrm{cm}$ are the maximum field and the length of the dipoles, and $\varepsilon$ is the energy of the electrons. For the energy of $\varepsilon = {200}\,\textrm{MeV}$ used in Fig. 4, the maximum steering angle is 2.6$^\circ$, leading to the initial array size of $\pm 5.4$ cm for the focusing distance of 1.2 m from the beamline. Because the bunches after the collimator still have a relatively wide spectrum, steering for larger angles also introduces additional beam divergence, as the deflection angle is inversely proportional to the electron energy. When using the array, this leads to the extended focus in the longitudinal direction. The properties of the focus are different in the *zx* and *zy* planes, owing to the asymmetry in both the initial beam and the beamline with respect to the transverse *x* and *y* directions.

### 3D Dose deposition calculation

The propagation characteristics of polychromatic electron beams generated by LPA within a water phantom are investigated by using realistic electron bunches generated from PIC simulations and filtered by the beamline. To validate the proposed design, 3D dose deposition calculations are conducted using Monte Carlo–based GEANT4 simulations. The edge of the phantom is located 1 m away from the end of the beamline, and it is assumed that the space between the beamline and the phantom is filled with air.

#### Dose distribution in homogeneous phantom

**Fig. 5:**
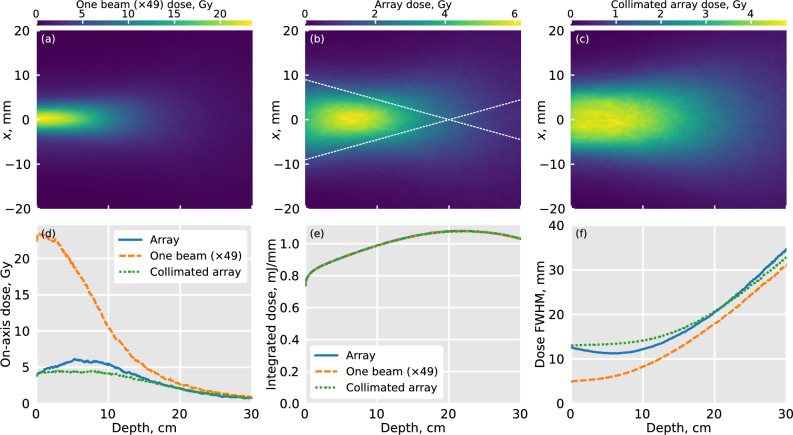
Dose distribution in the *zx* plane (*y* = 0) of the water phantom for the cases of (**a**) using one beam, (**b**) an array of beams for the beam corresponding to Fig. 1(d), (c) a $7 \times 7$ collimated array of beams with the same entrance size. The dashed lines in (**b**) show the trajectories of 200 MeV electrons from the beams with the maximum incidence angle in the absence of the phantom. The dependencies of (**d**) the on-axis dose (*x* = *y* = 0), (**e**) the dose integrated over transverse *xy* plane, (**f**) the transverse size (FWHM) of the dose distribution are presented.

As described in the previous section, we propose using an array of beams incident at different angles to create an optimal dose distribution and to demonstrate the capabilities of the start-to-end simulation approach. The dose distribution was analyzed through the calculation of the on-axis dose and integrated dose profiles. The on-axis dose provides a measure of the maximum dose delivered along the central axis of the beams, while the integrated dose evaluates the dose deposition as a function of depth within the phantom. Figure 5 shows the comparison between the dose deposition created by a $7 \times 7$ array of beams (see Fig. 4) incident at different angles in both *x* and *y* directions to one normally incident beam (repeated 49 times to achieve the same total dose absorbed by the phantom), as well as to a $7 \times 7$ collimated array of beams (each entering at normal incidence). The dose distribution from one collimated beam in Fig. 5(a) is very narrow around its entrance to the phantom. However, it quickly expands in the transverse direction with depth due to the scattering of electrons inside the phantom, leading to a drop in the on-axis dose in Fig. 5(d). This distribution is suboptimal because of a much larger entrance dose compared to the in-depth dose. However, when we use an array of beams incident at different angles, shown in Fig. 5(b), the peak on-axis dose moves deep inside the phantom, although it does not reach the same depth as the intended convergence point of all the beams in vacuum due to both the scattering and the dependence of the convergence point coordinate on the electron energy. For comparison, the dose from a similarly sized $7 \times 7$ collimated array, shown in Fig. 5, shows almost a flat profile of the on-axis dose and the width of the dose distribution for the depth up to 10 cm. At the same time, the distribution of the dose integrated over the transverse plane in Fig. 5(e) does not depend on the interaction geometry, as the incidence angles of the beams remain very small. The observed smoothness of the entrance dose shows that using a chosen $7\times 7$ array is enough to provide a sufficient overlap between doses deposited by different beams. With a lower number of beams in the array, the dose distribution profiles from individual beams would be distinguishable at the entrance, decreasing the ratio between the in-depth dose and the entrance dose.

**Fig. 6:**
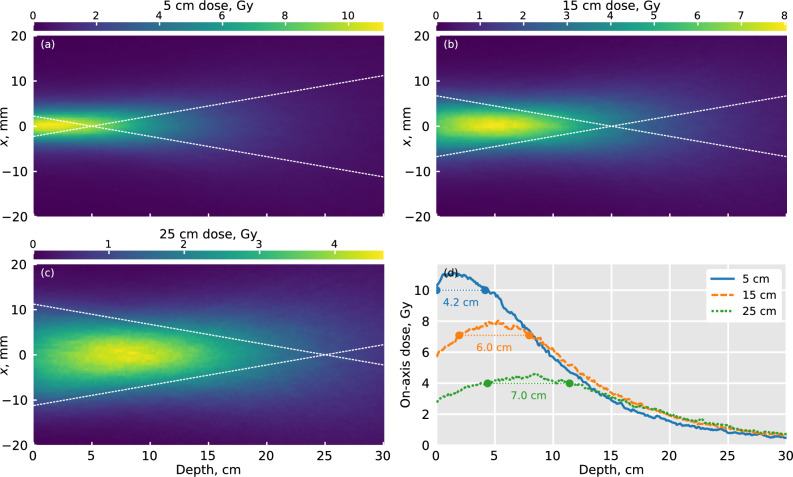
Dose distribution in the *zx* plane (*y* = 0) of the water phantom for different depths of intended beam array convergence (in vacuum): (**a**) 5 cm, (**b**) 15 cm, (**c**) 25 cm for the 200 MeV beam corresponding to Fig. 1(d). The dashed lines show the trajectories of 200 MeV electrons from the beams with the maximum incidence angle. (**d**) The dependencies of the on-axis (*x* = *y* = 0) dose on the depth for all three cases. The horizontal lines with values show the therapeutic range (width at 90% peak dose level).

By moving the point of convergence of the beams inside the phantom, we can achieve a different depth of the peak dose deposition (see Fig. 6). The figure demonstrates that for VHEE beams with the considered array geometry, the entrance dose is significantly lower relative to the peak dose delivered. Choosing the position of the convergence point provides flexibility in targeting tumours located at different depths while reducing the entrance dose. At the same time, the therapeutic range generally increases with the increase in depth, making the distribution less localized. The best localization is achieved by using the maximum angle of the array enabled by dipoles; using an array with a smaller angle would increase the therapeutic range and reduce the localization of the dose distribution. The ability to steer the beam with compact dipole magnets to generate array distributions is unique to electrons and constitutes an advantage over conventional photon and proton techniques.

**Fig. 7:**
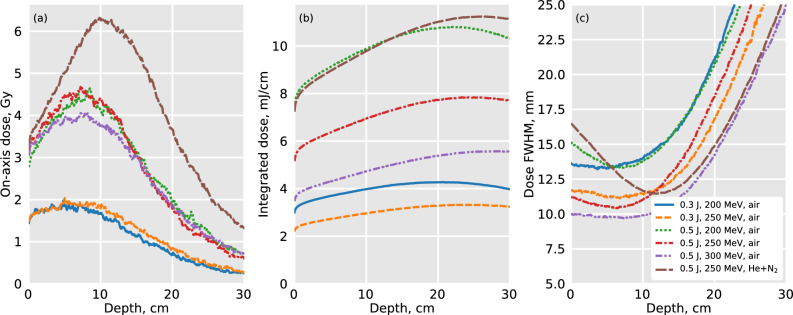
The dependencies of the (**a**) on-axis dose (*x* = *y* = 0), (**b**) the dose integrated over the transverse *xy* plane, and (c) the transverse size (FWHM) of the dose distribution for all beams from Fig. 1 when using a $7\times 7$ array with expected convergence (in vacuum) at the depth of 25 cm.

The comparison of the distributed dose properties when using arrays of different realistic beams from PIC simulations are shown in Fig. 7. In all cases, the dose distribution exhibits a similar behavior of having an on-axis peak deep inside the phantom, as shown in Fig. 7(a). The deepest dose peak is reached by a 200 MeV beam generated by a 0.5 J laser, as well as for a 250 MeV beam generated in a helium–nitrogen mixture, which can be explained by their comparatively lower relative energy spreads and thus smaller divergence added to the beams by the dipoles, leading to a more focused on-axis distribution with a deeper peak. This also corresponds to the fact that the transverse size of the dose distribution has a more clearly defined minimum in Fig. 7(c). For the other beams, the narrowest part of the dose distribution is spread over a larger depth. At the same time, the dose integrated over the transverse plane, shown in Fig. 7(b), is more uniform and is determined mostly by the spectrum of the beam, with its maximum being deeper for more energetic beams.

#### Dose distribution from multi-field irradiation

**Fig. 8:**
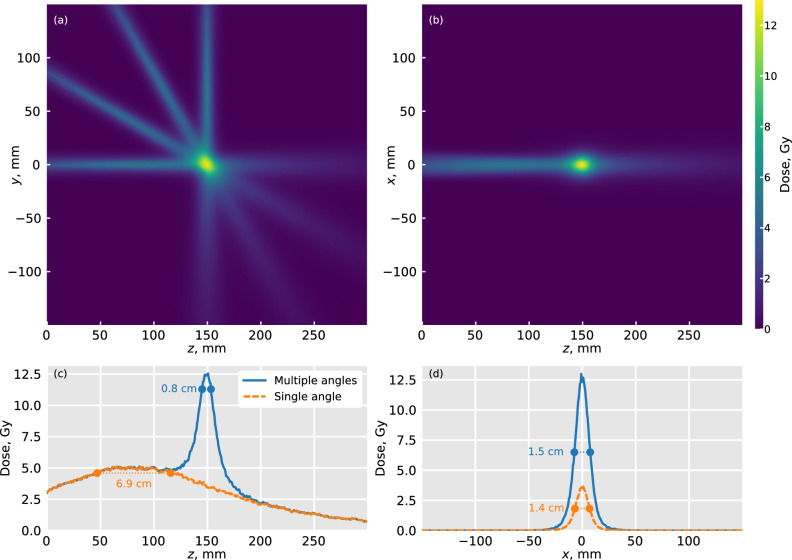
Multiple field irradiation in 0$^\circ$, 30$^\circ$, 60$^\circ$, and 90$^\circ$ angles at the isocentre of the phantom. (**a**) and (**b**) show the dose distribution in the *zy* ($x = 0$) and *zx* ($y = 0$) planes; (**c**) and (**d**) show the distribution along the *z* and *x* axes. For comparison, the dose distribution from a single array propagating along *z* is shown with a dashed line in (**c**) and (**d**). The horizontal lines with values in (**c**) show the therapeutic range (width at 90% peak dose level), in (**d**) FWHM of the distribution. The used beam corresponded to Fig. 1(d).

Four arrays of 49 collimated very high-energy electron (VHEE) beams were directed toward the isocenter^43^ with a focal point of 15 cm from the surface of the phantom measuring 30 cm $\times$ 30 cm$\times$ 30 cm (Fig. 8). To achieve comprehensive dose coverage, the beam arrays were delivered at incidence angles of 0$^\circ$, 30$^\circ$, 60$^\circ$, and 90$^\circ$. This multi-angle arrangement ensured the increased localization of the dose distribution and the reduction of the relative dose received by the surrounding volume which may represent healthy tissues. Each beam delivered a fraction of the total dose, and the angular distribution was arranged to conform the dose to the isocenter and target volume. In total, 196 beams converged at the isocenter, to evaluate the combined dose deposition profile. The used beams corresponded to Fig. 1(d) with the expected convergence in vacuum at the depth of 25 cm (similar to the 25 cm case in Fig. 6). Practically, this geometry can be achieved by creating each array using the transverse motion of the source and rotating the phantom to deliver several arrays from different angles.

Figure 8 demonstrates the dose distribution in the multifield irradiation setup. Both in planes *zy* and *zx*, shown in Fig. 8(a,b), we see significant localization of the deposited dose at the centre of the phantom. On the *z* axis corresponding to the propagation direction of one beam, shown in Fig. 8(c), the dose distribution has a distinct peak at the intended depth of 15 cm. The therapeutic volume (the volume in which more than 90% of the peak dose is delivered)^44^ is 180 $\hbox {mm}^3$, while the therapeutic range (the depth range at which more than 90% of the peak dose is delivered) is 8 mm. The peak dose delivered at the centre of the phantom is around 15 Gy, which is almost 4 times higher than the dose from a single angle at this position. At the same time, the shape of the distribution on the *x* axis which is transverse to the plane of the beam rotation, shown in Fig. 8(d), remains almost the same. The FWHM sizes of the dose distribution calculated in the *x*, *y* and *z* directions are 15 mm, 28 mm and 28 mm, respectively.

This method of dose delivery shows that combining the array with the rotation of the phantom can be used to effectively localize the delivered dose. As the array geometry significantly reduces the entrance dose compared to the in-depth dose (as shown in Fig. 5), even using several angular directions is enough to create a favorable dose distribution for reaching areas deep inside the phantom.

#### Dose distribution in heterogeneous phantom

The effect of tissue heterogeneity on dose distribution calculations presents a significant challenge in radiotherapy planning^19,45^. Variations in tissue density can substantially alter radiation beam paths and energy deposition patterns, particularly in proton-based radiotherapy techniques.

**Fig. 9:**
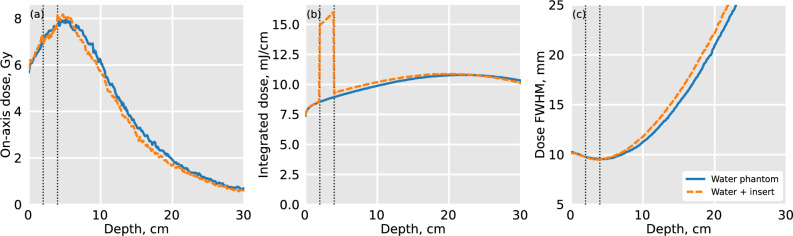
Comparison of dose distribution inside the water phantom and the heterogeneous phantom with a $7 \times 7$ array of beams corresponding to Fig. 1(d). The vertical lines show the boundaries of the 2-cm-thick bone insert.

The dose distribution in heterogeneous phantom with a 2-cm-thick bone insert with a center located at 3 cm from the surface of the phantom was measured using a $7\times 7$ array of 200 MeV beams corresponding to Fig. 1(d). The resulting on-axis and integrated dose distributions as well as the dependence of the dose transverse size on the depth are presented in Fig. 9. Our analysis of both the integrated dose distribution and on-axis dose measurements demonstrates minimal perturbation in the dose distribution pattern behind the bone insert.

This finding shows that using VHEE beams will not necessarily require complex correction algorithms to account for heterogeneities for dose delivery accuracy in anatomical regions containing tissues of varying densities. However, this will depend on the exact target location and requires additional research with more realistic models.

## Discussion and conclusions

Based on the PIC simulations with FBPIC and the GEANT4 simulation toolkit, we conducted start-to-end simulations of LPA-produced VHEE beam dose deposition for radiotherapy. Realistic beams were produced in a model of a gas jet simulated with FBPIC and propagated through a numerical model of the beamline based on a quadrupole doublet for filtering and collimating the beam as well as two magnetic dipoles for steering the beam and forming a beam array. The interaction of the beams with a homogeneous water phantom and a heterogeneous phantom with a 2 cm bone insert was then calculated with GEANT4.

Numerical measurements of 3D dose deposition were performed for an array of beams focusing at different incidence angles. The simulations show that using such an array can effectively move the peak of the dose distribution deep inside the phantom, thus making it possible to treat deep-seated tumours. The increased depth appears to be correlated with a reduction in energy spread. Therefore, optimizing for a narrower energy spread while keeping the high total charge will be an important experimental task.

The use of multi-field irradiation with several arrays of beams coming from different angles around the phantom further demonstrated the ability to localize the peak dose and prevent damage to surrounding tissues. The dose deposition in heterogeneous phantoms with a 2 cm bone insert showed low sensitivity of the dose distribution to inhomogeneities, possibly indicating that complex correction algorithms might not be required for effective treatment. While known heterogeneities from planning imaging can be incorporated into treatment plans, this low sensitivity might be advantageous in cases when patient physiology changes between treatment fractions or when treating sites with significant organ motion.

The results confirm that LPA-produced VHEE beams are a promising alternative to conventional radiotherapy techniques, particularly for treating tumours in challenging locations or in sensitive patient populations. However, more detailed research and comparison to alternative dose delivery schemes is needed to investigate clinical application of the proposed VHEE delivery scheme in simulations and to develop treatment plans.

Compared to more simplified approaches based on experimentally informed models of the beam, the start-to-end simulation workflow takes into account the full complexity of the LPA-generated beams’ phasespace and spectrum and allows for studying the dependencies of the dose distribution on the LPA device parameters and the beamline design. Future research can explore other injection techniques, compare different beamline designs and interaction with more complex phantoms, or measure the sensitivity of the deposited dose distribution to the jitter in the gas density profile and laser pulse parameters, reflecting the stability considerations of LPA devices. The beamline could also be simulated inside GEANT4 (instead of using a simplified model that only calculates the electron motion) to calculate the activation of the beamline elements as well as the irradiation of the phantom with secondary particles, such as bremsstrahlung from the electron interaction with the collimator.

## Methods

### PIC simulations

We perform PIC simulations using angular mode decomposition with FBPIC^39^. The laser pulse with the central wavelength of 800 nm has a Gaussian temporal and transverse profiles at the focus, the duration of 25 fs (power FWHM) and is focused to a spot size of 12 $\upmu$m (radius at $1/e^2$ intensity). Two pulse energies of 0.3 J and 0.5 J are considered. The laser is initialized before the entrance to the gas jet and out of focus, with the longitudinal coordinate of its focus relative to the jet being a variable parameter.

The gas jet is modeled by a trapezoidal profile with 0.2-mm-long up- and down-ramps to simulate the transition to vacuum and variable length of the plateau of variable density. The gas composition consists of 80% nitrogen and 20% oxygen to model air. As simulations rely on ionization injection, they consider nitrogen and oxygen preionized only to levels 4 and 5, respectively. Further ionization of lower shells is enabled by the tunnel field ionization module in FBPIC, allowing us to simulate ionization injection.

**Table 1:** Optimized PIC simulation parameters for the beams shown in Fig. 1. The density value corresponds to the plasma density assuming partial ionization of nitrogen and oxygen to levels 5 and 6, respectively, and full ionization of helium.

Case	Density, cm$^{-3}$	Plateau length, mm	Longitudinal laser focus position, mm
Case 1: 0.3 J, 200 MeV, air	$8.70 \times 10^{18}$	2.15	2.02
Case 2: 0.3 J, 250 MeV, air	$6.61 \times 10^{18}$	2.40	1.34
Case 3: 0.5 J, 200 MeV, air	$7.64 \times 10^{18}$	2.47	0.62
Case 4: 0.5 J, 250 MeV, air	$7.27 \times 10^{18}$	2.52	0.27
Case 5: 0.5 J, 300 MeV, air	$5.93 \times 10^{18}$	3.32	2.56
Case 6: 0.5 J, 250 MeV, He+N$_2$	$7.67 \times 10^{18}$	2.30	2.25

The plasma density, the length of the plateau, and the longitudinal position of the focus are considered free parameters and are given to a genetic algorithm based on PyGAD^46^ which optimizes these three parameters to provide the electron beam with the highest possible charge in the desirable energy range. The optimized parameters are shown in Table 1. One of the simulations was performed with a helium–nitrogen mixture instead of air. In this case, helium was assumed to be fully preionized, and the share of nitrogen was added as an optimization parameter. The optimal mixture was found to be 95% helium and 5% nitrogen (N$_2$).

In the simulations, we used a simulation box with step sizes of 0.02 $\upmu$m and 0.16 $\upmu$m in the longitudinal and transverse directions, respectively, with 3 azimuthal modes, and the time step *ct* equal to the longitudinal step size. The size of the box in the longitudinal direction was equal to 65.5 $\upmu$m (3273 cells), while the transverse size was no less than 42 $\upmu$m (262 cells), but could also be automatically adjusted to ensure the initial not fully focused pulse fits into the simulation box. Open boundary conditions were used on the radial boundary. Preionized atoms and electrons were initialized with $48 = 2 \text {\,(long.)} \times 2\text {\,(trans.)}\times 12\text {\,(azim.)}$ particles per cell. To accelerate the simulations, the Lorentz boost technique with the Lorentz factor $\gamma _\textrm{boost} = 4$ was used^47^. Each simulation was performed on 3 GPUs with parallelization between the GPUs through the Message Passing Interface (MPI).

### Beamline simulations

All electrons with energies above 5 MeV from the final iteration of the FBPIC output are passed to the simulated beamline corresponding to Fig. 2. Propagation of the beam through quadrupoles is modelled by Wake-T^48^ simulations, assuming the quadrupoles have a uniform longitudinal distribution of the magnetic field. The quadrupoles have an inner radius of 3 mm, and all electrons which exceed this radius both at the entrance and the exit of the quadrupoles are filtered out. Propagation in vacuum sections is calculated analytically (space charge effects are neglected). The collimator is simulated by filtering out all electrons with the distance from the beamline axis above 1 mm at its coordinate. The magnetic dipoles are simulated by calculating analytical solutions of electron motion in the relativistic limit $\gamma \gg 1$ assuming constant magnetic field inside the dipoles.

### Quadrupole position optimization

Magnetic quadrupoles are electron beam transport line elements which provide focusing in one axis while defocusing in the other. The magnetic field of a quadrupole is given by

1 $$\begin{aligned} {\textbf {B}} = G \nabla {(x y)} = (G y, G x, 0), \end{aligned}$$

where $G = {545}\,\textrm{T/m}$ is the magnetic field gradient.

The focal length of a doublet quadrupole lens is given by

2 $$\begin{aligned} f = \frac{\gamma mc^2}{ec} \frac{1}{\int G dz} = \frac{E_\textrm{kin}}{ec} \frac{1}{GL} \end{aligned}$$

where *L* is the quadrupole length, $E_\textrm{kin}$ is the electron energy, *e* is the elementary charge, and *c* is the speed of light. The imaging condition for a lens with focal length *f* relates the source position *s* and image position *u* as:

3 $$\begin{aligned} \frac{1}{f} = \frac{1}{s} + \frac{1}{u}, \quad u=\frac{sf}{s-f} \end{aligned}$$

When optimized, a quadrupole doublet can achieve beam collimation with the second lens positioned at a specific distance from the first lens’s imaging position.The optimal configuration occurs when $z_1 = 2f_1$, $f_1 = (4/3)f_2$, and $z_2 = (8/3)f_1$, resulting in a beam size of $\sigma _x = (4/3) f_1 \theta _x$, $\sigma _y = 4 f_1 \theta _y$ after collimation, where $\theta _x$ and $\theta _y$ are initial angles of divergence. The beam from the LPA stage usually is more divergent in the laser polarization direction, so this direction is chosen to correspond to the *x* axis so that $\theta _x> \theta _y$. In this case, the resulting collimated beam will have a more circular shape than before collimation.

**Table 2:** Beamline parameters: focal distances $f_1$ and $f_2$ and positions $z_1$ and $z_2$ of the quadrupoles, the positions of the collimator $z_\textrm{coll}$ for different beams from Fig. 1.

Case	$f_1$, cm	$f_2$, cm	$z_1$, cm	$z_2$, cm	$z_\textrm{coll}$, cm
Case 1: 0.3 J, 200 MeV, air	6.75	9.00	13.50	18.01	33.01
Case 2: 0.3 J, 250 MeV, air	8.44	11.25	16.87	22.50	37.50
Case 3: 0.5 J, 200 MeV, air	6.75	9.00	13.50	18.01	33.01
Case 4: 0.5 J, 250 MeV, air	8.44	11.25	16.87	22.50	37.50
Case 5: 0.5 J, 300 MeV, air	10.12	13.49	20.24	26.99	41.99
Case 6: 0.5 J, 250 MeV, He+N$_2$	8.44	11.25	16.87	22.50	37.50

Assuming that $G_1 = G_2$, the optimization criterion $f_1 = (4/3)f_2$ requires that $L_1 = (4/3)L_2$, which is why quadrupoles are taken to have the thicknesses of $L_1={2}\,\textrm{cm}$ and $L_2={1.5}\,\textrm{cm}$. Due to the finite thickness of quadrupoles in beamline simulations, the thin lens approximation used in Eqs. (2)–(3) does not provide ideal collimation for the target energy. To achieve better collimation, an empirically corrected energy of $E_\textrm{kin}'=1.1 E_\textrm{kin}$ is used in Eq. (2). So for the 200 MeV, 250 MeV, and 300 MeV beams, the values of $E_\textrm{kin}'$ used to calculate the beamline parameters are 220 MeV, 275 MeV, and 330 MeV, respectively. The corresponding focal lengths $f_{1,2}$ and the positions $z_{1,2}$ of quadrupoles depend on the corrected target energy $E_\textrm{kin}'$ of the beam and are shown in Table 2.

### GEANT4 simulations

The Monte Carlo-based GEANT4 toolkit is used to simulate 3D dose deposition. GEANT4 provides various physics lists to simulate the passage of particles. In our studies, we use the G4EmStandardPhysics_option4 physics list for dose calculations, as it includes all physical processes involved during electron interaction with matter in the required energy range^49^. This physics list is widely recommended and used by medical physicists for dose deposition calculations^50^.

The beam data from the end of beamline simulations are saved to the disk and read by GEANT4 simulations to initialize the primary particle source through a custom G4VUserPrimaryGeneratorAction interface. Each primary event corresponds to one macroparticle from the imported distribution with its weight directly corresponding to the weight of the macroparticle.

To quantitatively assess the dose distribution characteristics in both homogeneous and heterogeneous media for the polychromatic nature of beams, we use two phantom setups. For homogeneous media testing, we employ a rectangular water phantom (G4_WATER, 30 cm $\times$ 30 cm $\times$ 30 cm, density of 1 $\textrm{g}/\textrm{cm}^3$) placed in an air-filled (G4_AIR) world. The shape of the phantom has a negligible influence on the dose distribution for the small beam incidence angles ($<{3}{^\circ }$) considered. The phantom was divided into 500 $\times$ 500 $\times$ 300 voxels (with the last *z* direction corresponding to beam propagation) to enable precise dosimetric analysis. For multi-field irradiation, the phantom is divided into 300 $\times$ 300 $\times$ 300 voxels. For heterogeneous media testing, we use a water phantom of a 10 cm $\times$ 10 cm $\times$ 30 cm size with a 2-cm-thick G4_BONE_COMPACT_ICRU insert with a density of 1.85 $\textrm{g}/\textrm{cm}^3$ centered at 3 cm below the phantom surface. The phantom is divided into 500 $\times$ 500 $\times$ 300 voxels. The production cut in the phantom is set to 0.1 mm. The energy deposited in each voxel is normalized to the mass of the voxel to obtain the dose in Gy. The on-axis dose is calculated from the central line of voxels along the beam axis.

## Data Availability

The data is available upon reasonable request to the authors.
